# Examination of resistance of various methods of pulmonary vessel occlusion to hydrostatic pressure

**DOI:** 10.1007/s12055-023-01505-8

**Published:** 2023-04-15

**Authors:** Darko Gajić, Małgorzata Wojtyś, Norbert Wójcik, Bogumił Maciąg, Kajetan Kiełbowski, Janusz Wójcik, Tomasz Grodzki

**Affiliations:** Department of Thoracic Surgery and Transplantation, Pomeranian Medical University, Alfreda Sokołowskiego 11, 70-891 Szczecin, Poland

**Keywords:** Bursting pressure, Vessel occlusion, Ligation, Clips, Endostapler

## Abstract

**Objective:**

The aim of this study was to compare pressure resistance of the most common methods of vessel occlusion during thoracic surgical procedures: ligations, clips, and vascular endostaplers.

**Methods:**

Pulmonary vessels were obtained during routine thoracic surgeries. A ball-tipped cannula was inserted through an opening in the side wall and secured with a linen ligature from slipping out. Subsequently, saline was infused into the vessel. We recorded the pressure on which a leakage occurred.

**Results:**

A total of 65 vessels, divided between 3 groups, were enrolled in the study. In the endostaplers group, median bursting pressures were 262.5 mmHg and 300 mmHg for arteries and veins, respectively. In the case of clips, it was over 750 mmHg in both types of the vessels. The same results were observed in the ligation group. Minimal bursting pressures in endostapler occlusion were 187.5 mmHg and 225 mmHg in arteries and veins, respectively. In the case of clips, it was 600 mmHg for arteries and 675 mmHg for veins. A total of 525 mmHg (arteries) and 750 mmHg (veins) were the minimal leaking values observed in vessels occluded with ligations. Comparative analysis showed statistically significant differences in endostapler-clips and endostapler-ligations pairs (*p* < 0.001). There were no differences between clips and ligations.

**Conclusions:**

The examined methods are capable of occluding pulmonary vessels under physiological conditions. Furthermore, ligations and clips are resistant to pressures highly exceeding physiological values.

## Introduction

One of the key maneuvers during the resection of a pulmonary lobe or an entire lung is to occlude pulmonary vessels supplying the resected pulmonary parenchyma. A surgeon is the one to decide which method shall be applied for vein or artery occlusion. Since minimally invasive surgeries have become more popular, various methods are available, each having advantages and disadvantages. Ligations, clips, and endostaplers are the most common methods used in thoracic surgeries. Ligation is the oldest method and has an established position in almost every open surgery. Furthermore, it is possible to apply ligation in minimally invasive surgeries. Special tools that stabilized the intracorporeal knots were used. However, this is a less popular method which gives way to staplers and clips [1–3]. Endostaplers constitute the basic method of supplying vessels during minimally invasive surgeries. They allow for proximal and distal occlusion with a simultaneous intersection. Therefore, the simplicity of using endostaplers represents a significant asset compared to clips and ligation. The aim of this study was to examine and compare the resistance of the most frequent methods of pulmonary vessel occlusion to hydrostatic pressure.

## Material and methods

**Fig. 1:**
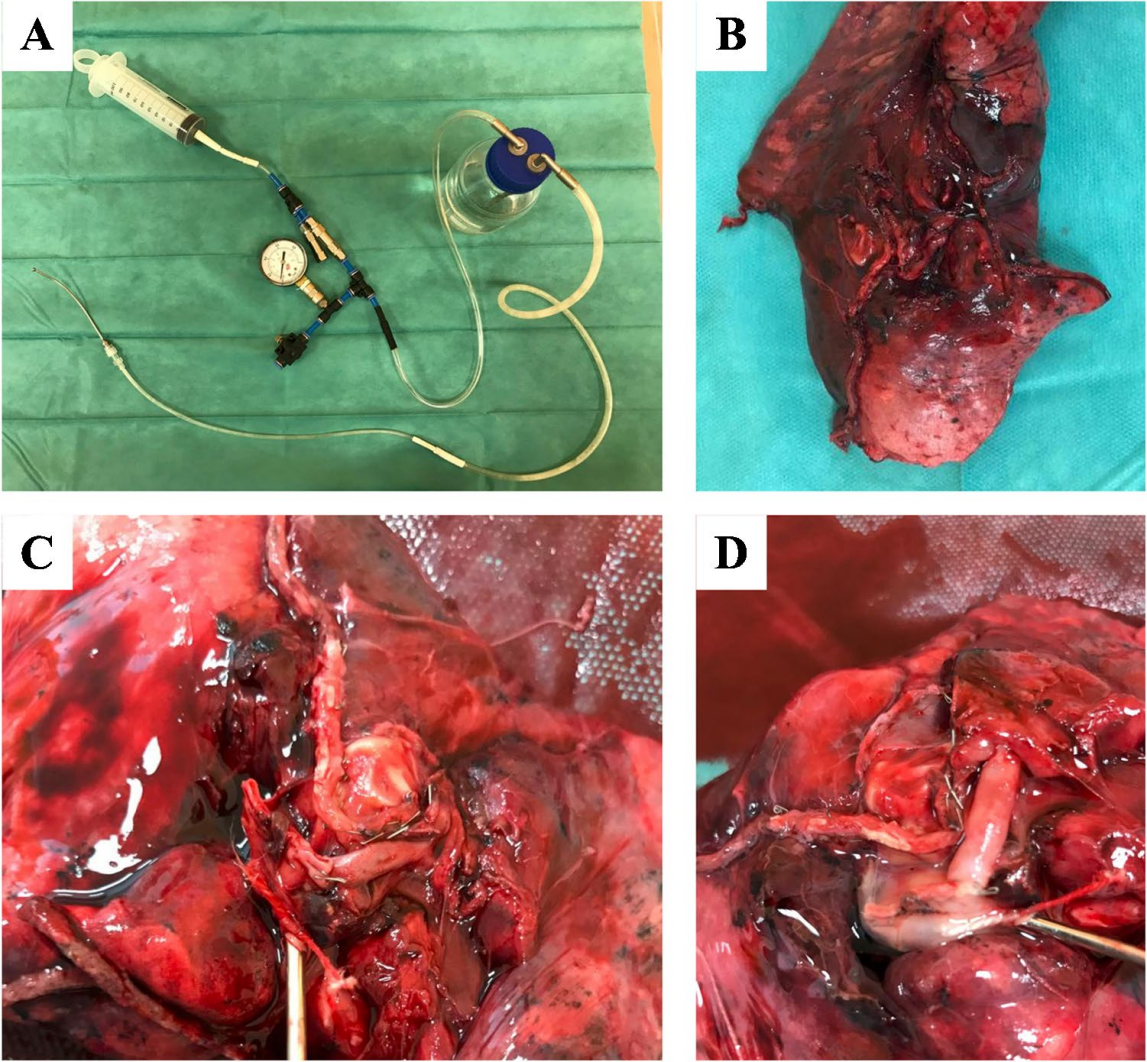
**A**: A device with a metal cannula and piston gauge, which was used in the present study; **B**: Surgical specimen; **C**, **D**: A cannula was inserted in the vessel and the pressure was increased until it broke the vessel, occlusion, or until the maximum measurement was obtained

Pulmonary vessels of 33 specimens collected from the patients’ undergoing surgeries at the Department of Thoracic Surgery and Transplantation were examined. Pulmonary arteries and veins of various sizes were divided between 3 groups. The first group involved vessels closed with the vascular endostaplers. Endo GIA Tri-Staple™ devices were used in the study. The second group was composed of arteries and veins occluded with the use of linen ligatures (typical technique — two proximal ligations and one distal ligation). The third group involved vessels secured with Click’aV Plus™ polymer clips. Measurements were performed ex vivo, after resection of the lung or lobe. A piston gauge was used to generate hydrostatic pressure. It allowed to obtain a controlled and measurable pressure inside the selected vessel. A ball-tipped cannula was inserted through an opening in the side wall and secured with a linen ligature from slipping out. Subsequently, saline was infused into the vessel. The pressure was increased until it broke the vessel, occlusion, or until the maximum measurement was obtained (approximately 750 mmHg) (Fig. 1). In vessels supplied with staplers, the results were noted after the leakage in the staple line was observed.

### Exclusion criteria

Patients with collagenases, pulmonary hypertension, or any other disease that could affect the resistance of the vessel’s wall were not included. Furthermore, cancer-infiltrated arteries were not evaluated.

### Statistical analysis

Mean, median, and standard deviation (SD) for each of the methods have been calculated. The Kruskal-Wallis test and Dunn test were used to compare the obtained values. Statistical analyses were performed using SPSS software ver. 25.0 (IBM, Armonk, NY, USA). A result was considered statistically significant if “p” value was  < 0.05.

## Results

A total of 65 vessels were analyzed. The majority of the included vessels were lobar arteries and veins (Table 1). The use of the main pulmonary arteries was incidental. Results in mmHg are presented in Table 2. In the group involving endostaplers (*n* = 22 each), medians of occlusion resistance in arteries and veins were 262 mmHg and 300 mmHg, respectively. The minimum value at which leakage was observed was 187.5 mmHg in arteries and 225 mmHg in veins. The rupture of a free vessel wall during the examination has not been observed. The second group included vessels occluded with linen ligatures (*n* = 20 ). In both types of vessels, the median rupture value exceeded 750 mmHg. Minimum leaking pressure values during the examination were as follows: 525 mmHg for arteries and over 750 mmHg for veins. One rupture occurred in the artery in the area where the ligation was placed (525 mmHg). In the group with polymer clips, one vessel was damaged, and this resulted in exclusion from the study. In total, 11 veins and 12 arteries were further examined. Median bursting pressure for arterial and venous vessels was greater than 750 mmHg. The minimum values obtained were 600 mmHg for arteries (rupture of the vessel’s free wall) and 675 mmHg for veins (rupture in the clip’s arm line). Furthermore, we have observed that the most diversified values were in the endostapler group (SD_V_ = 85,96; SD_A_ = 99,34). SD calculated for the group of clips (SD_A_ = 58,39; SD_V_ = 22,61) and ligations (SD_A_ = 70,82; SD_V_ = ,0000) were significantly lower. Kruskal–Wallis test demonstrated that particular measurements of veins and arteries were significantly diversified within the analyzed groups. The exact Dunn pair test demonstrated that in each analyzed case, the statistically significant differences refer to endo staplers-ligation and endo staplers-clips pairs (*p* < 0.001). No statistically significant differences were demonstrated between ligations and clips.

**Table 1:** Specimens used in the study

Specimens	N
Right upper lobe	10
Right middle lobe	2
Right lower lobe	6
Right middle and lower lobes	2
Right lung	1
Left upper lobe	2
Left lower lobe	10
Overall	33

**Table 2:** Statistical evaluation of the efficacy of endostaplers, ligatures, and clips

Group		Vein (mmHg)	Artery (mmHg)
Endostaplers Artery *n* = 11 Vein *n* = 11	Mean	323.86	289.77
	Median	300	262.5
	Standard deviation	85.96	99.34
	Minimum	225	187.5
	Maximum	487.5	562.5
Ligation Artery *n* = 10 Vein *n* = 10	Mean	750	723.75
	Median	750	750
	Standard deviation	.00	70.82
	Minimum	750	525
	Maximum	750	750
Clips Artery *n* = 12 Vein *n* = 11	Mean	743.18	725
	Median	750	750
	Standard deviation	22.61	58.39
	Minimum	675	600
	Maximum	750	750
In total Artery *n* = 33 Vein *n* = 32	Mean	601.17	579.55
	Median	750	750
	Standard deviation	210.09	221.28
	Minimum	225	187.5
	Maximum	750	750
*p* (Kruskal Wallis test)		<0.001	<0.001
Dunn test (artery)			
Endostapler vs ligation		<0.001	
Endostapler vs clips		<0.001	
Ligation vs clips		1	
Dunn test (vein)			
Endostapler vs ligation		<0.001	
Endostapler vs clips		<0.001	
Ligation vs clips		1	

## Discussion

Progress observed in minimally invasive surgery involves the development of new methods of vessel occlusion. They should be characterized by resistance, simplicity of application, and relatively low price. This study aimed to compare the methods most often used nowadays. According to the results obtained, endostaplers were the least resistant to ex vivo–generated hydrostatic pressure. Statistically significant higher values were obtained in the case of clips and ligations. Moreover, these methods did not differ significantly. In addition, higher mean pressure values for occlusion were obtained in veins than in arteries in every method evaluated. Nevertheless, these results were not statistically significant. These observations could be attributed to the histological structure and vulnerability of the vessels’ walls. The effectiveness of hemostasis maintenance with vascular ligation depends on the technique chosen by the operator. Therefore, to minimize the risk of a human mistake and potentially dangerous hemorrhage, two ligatures are applied for the proximal part of the stump in pulmonary vessels in both lobectomies and pneumonectomies. The pressure generated in the vessels occluded with ligatures significantly exceeded physiological conditions (the lowest pressure at which leakage was observed was 525 mmHg). In a separate study by Lim et al., the authors compared both veins and arteries collected from rabbits occluded with the use of ligatures, and similar values were observed [4]. Moreover, Rajbabu et al. have not observed leakage from the vessel occluded with ligatures with values lower than 300 mmHg (maximum pressure used during their experiment) [5]. Therefore, the above studies confirm that ligation is a safe method for occluding supplying vessels. It can be used to occlude vessels of various diameters, from large pulmonary arteries to small segmental vessels. Pressure values obtained with the use of Endo GIA Tri-Staple™ endostaplers were significantly lower (arteries = 262.5 mmHg: veins = 300 mmHg) than in the case of linen ligatures and Click’aV Plus™ polymer clips. Similar results were observed by Joseph et al., who used swine renal arteries [6]. The authors observed leakage in the staple line in 50% of cases with an average pressure of 273 mmHg (237–322 mmHg). There was no leakage in vessels closed with polymer clips and ligatures with values below 800 mmHg [6]. Other studies also report high pressures obtained in vessels occluded with clips and ligatures (300–1800 mmHg) [2, 7–9]. Caution is required while using a stapler as improper fastening may reduce its effectiveness [10, 11]. This is crucial especially in minimally invasive techniques, where straightened access increases the risk of excessive pressure or vessel torsion. This may result in dangerous and difficult-to-stop hemorrhage requiring conversion to thoracotomy. Staplers can be used for the occlusion of vessels of various diameters. Furthermore, they are used to occlude bronchi and to resect pulmonary parenchyma. In the present study, similar pressures were obtained in vessels occluded with clips and ligatures. Lim et al. achieved the pressure of 548.02 ± 277.71 mmHg in the case of arteries occluded with Hem-o-Lok clips, while significantly lower values were obtained in the case of veins (79.84 ± 47.04 mmHg) [4]. However, such discrepancy in the results obtained is difficult to explain. It could result from a different structure of clip serration. In the conducted study, we have not observed clips sliding off the vessel, which can indicate good stability in the tissue. In a study by Saki et al., the clips did not slide off with the strength below 11.8 N which makes them safe in the situation of accidental pulling [12]. While applying a clip, it is important to avoid slamming the wall of the vessel in the latch, as it can lead to detachment, and consequently, hard-to-control hemorrhage. Various sizes of clips are available in the market. Unfortunately, for the time being, they are not big enough to be applied to the left or right pulmonary artery or upper pulmonary vein. However, Lucchi et al. supplied pulmonary veins with the largest clips with good results [13]. The described methods of vessel occlusion complement each other in clinical practice. Because of the low cost and habits of the operators, ligatures will constitute the basic tool used for pulmonary vessel management in open surgical procedures for a long time. It should be noted that pressure values on which leakage was observed were significantly higher than the physiological pressure within both pulmonary and systemic circulation. However, in patients with high, uncontrolled blood pressure surges, polymer clips and ligatures seem to be safer.

## Limitations

This study cannot be considered without certain limitations. Firstly, static pressure was used in the measurements. Physiological pulsatile pressure present in the human organism can be more destructive for the occlusion. Secondly, the majority of arteries used in this study were lobar arteries. The main pulmonary arteries were incidental. Nevertheless, we have not compared these two types of vessels.

## Conclusions

The evaluated methods of occlusion are safely used in clinical practice. Nevertheless, as demonstrated in this study, every occlusion technique has different pressure resistance. Occlusions made with ligatures and clips were more resistant to high pressures than the ones created with the use of staplers. Therefore, staplers need to be used with caution, taking care to not cause excessive vessel tension or torsion. Furthermore, staplers showed the highest variability of results. Ligatures and polymer clips show similarly high resistance to high pressure and their use is justified from the economic point of view. Examined methods are sufficient for the physiological pressure values of systemic and pulmonary circulation.

## Data Availability

The data that support the findings of this study are available from the first author, Darko Gajić, upon reasonable request at d_gajic@outlook.com.
